# Endolysin selectively kills *Gardnerella* ex vivo in vaginal samples from women with bacterial vaginosis

**DOI:** 10.1038/s41522-025-00764-0

**Published:** 2025-08-12

**Authors:** Lenka Podpera Tisakova, Timo Schwebs, Rocío Berdaguer, Marina von Freyberg, Philipp Foessleitner, Ann-Katrin Kieninger, Albina Poljak, Lorenzo Corsini, Alex Farr

**Affiliations:** 1 BioNTech R&D (Austria) GmbH, Vienna, Austria; 2 Medical University of Vienna, Department of Obstetrics and Gynaecology, Division of Obstetrics and Feto-Maternal Medicine, Vienna, Austria; 3 Vincent Center for Reproductive Biology, Massachusetts General Hospital, Harvard Medical School, Boston, MA USA

**Keywords:** Antimicrobials, Bacteriology

## Abstract

Current treatments for bacterial vaginosis (BV) often result in recurrent disease. *Gardnerella*, a key player in BV pathogenesis, forms biofilms on vaginal epithelial cells. Recombinant endolysins have shown to specifically kill *Gardnerella*, but not commensal lactobacilli, in vitro. This study evaluated the pharmacodynamics of BNT331-endolysin (BNT331-EL) on vaginal samples from 49 women with BV (Nugent score ≥7, Amsel criteria, and clue cells). Whole genome sequencing confirmed BV-associated community state types IV-B and III, with *Gardnerella* dominating in 53% of samples and present in 86%. Ex vivo treatment with BNT331-EL reduced viable *Gardnerella* by ≥94% at 20–50 µg/mL over 19 h. *L. iners* was reduced by an average of 92% across samples, while *L. crispatus* proliferated where present in substantial amounts. Endolysin treatment effectively disrupted *Gardnerella* biofilms and reduced viable bacterial load in a time- and concentration-dependent manner. These results informed the definition of the treatment dose for a first-in-human trial with BNT331-EL.

## Introduction

Bacterial vaginosis (BV) is the most common vaginal infection in women of child-bearing age^1^ and is characterized by a vaginal microbiome imbalance. The vaginal microbiome can be categorized into community state types (CSTs)^2^. BV is most frequently associated with CST IV, followed by CST III. CST IV-B is characterized by a diverse group of anaerobes such as *Gardnerella*, *Prevotella*, *Atopobium*, and *Sneathia*, and a very low abundance of *Lactobacillus*. CST III is characterized by a high abundance of *Lactobacillus iners* (*L. iners*), which is less protective against vaginal dysbiosis than other lactobacilli and is associated with shifting from the healthy into the BV state^3,4^. The role of *Gardnerella* in BV pathogenesis is subject of ongoing debate, but is often considered as key in the etiology^5–8^. Notably, *Gardnerella* initiates biofilm formation on vaginal epithelial cells, which provides a scaffold for other pathogens to proliferate^4,5,9–11^.

There is an unmet need for new BV therapies, as evidenced by its high prevalence (20-30% of all women^12,13^), profound impact on the health and well-being of women, fetuses and newborns^14,15^, and the high recurrence rate after standard of care (SOC) treatments^10,16^. Broad spectrum antibiotics, such as metronidazole (MTZ), provide quick relief to a subgroup of patients, but frequently result in recurrence (up to 60%) and the development of bacterial resistance^10,17–19^. Failure of antibiotics to eradicate the biofilm may contribute to the high relapse rate^9,18,20,21^. Endolysins are under investigation as potential alternatives to antibiotics due to their ability to specifically target the cell wall of individual bacterial genera, biofilm penetration, and low propensity for development of resistance^22^. BNT331-EL is an engineered, domain-swapped variant of a *Gardnerella* prophage-encoded endolysin that differs by one amino acid from a previously reported BioNTech-developed variant, PM-477^23^. Both variants are equivalent in their activity, however, BNT331-EL is advantageous in recombinant expression and stability.

We previously reported that PM-477 specifically kills planktonic *Gardnerella*, dissolves *Gardnerella*-dominated biofilms without harming beneficial *Lactobacillus*^23,24^ and is active against MTZ-resistant *Gardnerella* strains in vitro^25^. Establishing an in vivo BV/*Gardnerella* infection model is challenging due to the distinct human vaginal environment in terms of pH and microbiome^26–28^. Therefore, an arguably more relevant preclinical test system are vaginal swabs or vaginal fluid from women with BV for ex vivo treatment, which comprise critical disease elements (biofilm, relevant microbiome and pH, vaginal fluid matrix). Here, we characterized the pharmacodynamics of BNT331-EL by measuring its activity over time and selectivity on *Gardnerella* in vaginal samples ex vivo.

## Methods

### Study participant enrollment, sample collection and processing

Participants were diagnosed with BV either at the study site (Medical University of Vienna) or by an outpatient gynecologist and centrally confirmed. Inclusion criteria were age of ≥18, Nugent score ≥7 and 3/4 Amsel criteria with mandatory presence of clue cells. Exclusion criteria were Candidosis, Gonorrhea, Trichomoniasis, ongoing systemic or vaginal antimicrobial therapy, or any other kind of vaginally applied medication, probiotics or medicinal products. Two vaginal swabs (Copan eSwab 480CE with Amies medium) and neat vaginal fluid were collected from each enrolled participant. Samples were immediately transported to the external experimental lab (BioNTech R&D Austria) at 4 °C. 500 μL saline (B Braun NaCl 0.9% Rinse, Fisher Scientific) was added to the fluid sample, vortexed and aliquoted into cryovials. Two vaginal swab eluates were pooled, supplemented with 400 μL New York City Broth III pH 5.5 + 10% horse serum (Thermo Scientific), and aliquoted into cryovials. Samples were frozen at −80 °C.

### Genomic DNA extraction for whole genome sequencing (WGS)

Genomic DNA (gDNA) was extracted using the High Pure PCR Template Preparation Kit (Roche) with some changes. A total of 500 µL of swab samples were centrifuged at 8000 × *g*, the cell pellet washed with 500 µL saline and resuspended in 400 µL Tissue Lysis buffer with 100 µg lysozyme and incubated for 1 h at 37 °C. A total of 400 µL Binding buffer, 8 µL RNase A (17,500 U/mL, Qiagen) and 80 µL Proteinase K were added, and the samples incubated for 10 min at 70 °C prior to addition of 200 µL isopropanol and proceeding according to the instructions. DNA concentration was measured on a NanoDrop instrument.

Shotgun WGS (30 M total DNA sequencing), analysis and assignment of CSTs using the vaginal community state type Nearest Centroid classifier algorithm^29^ was performed by CosmosID (USA).

### Ex vivo treatment of participant samples

PM-477 and BNT331-EL were recombinantly expressed and purified as previously described^25^ and stored in MES buffer (50 mM MES pH 5.5, 200 mM NaCl, 8 mM MgSO_4_). After thawing on ice, baseline samples were directly used for downstream procedures (Fluorescence in situ hybridization [FISH] or viability quantitative PCR [qPCR]) without incubation. For treatments, 55-114 µL of each sample was either mixed with endolysin to yield a final concentration of 5, 20, 50, or 500 µg/mL, or with the same volume of MES buffer in a 96-U-well plate (Sigma Aldrich). Reaction plates were statically incubated for 3 h or 19 h at 37 °C under anaerobic conditions in tightly sealed plastic boxes with an anaerobic atmosphere generation bag (Fisher Scientific) and an anaerobic indicator. After mixing the samples, a 20 µL aliquot was used for FISH. The remainder was used for viability qPCR. Treatment reactions involving participant samples P031-P054 were supplemented to a final concentration of 1x NYC pH 5.5 with 10% horse serum.

### FISH microscopy

FISH was performed as described in Swidsinski et al. with some modifications^30^. In brief, the samples were fixed with 180 µL Carnoy’s solution (absolute ethanol, chloroform, acetic acid in ratio (v/v) 6:3:1). Twenty µL of the fixed samples were transferred onto a ~1.5 cm-diameter field marked on SuperFrost slides (Thermo Scientific) with a PAP pen (Agilent Technologies) and dried at 50 °C for 30 min. For hybridization, 0.5 µL of 50 ng/µL DNA hybridization probes (Gard662-5Alexa488 for *Gardnerella*, Eub338-5Alexa594 for Eubacteria, both from IDT) in 900 mM NaCl, 20 mM Tris-HCl pH 7.4, 0.005% SDS was added and incubated in a humid atmosphere for 60 min at 50 °C. The slides were rinsed with prewarmed (50 °C) sterile water and washing buffer (900 mM NaCl, 20 mM Tris-HCl pH 7.4), followed by two washes in deionized water. Human cells were stained with 200 μL of 1 μg/mL DAPI (4’,6-diamidino-2-phenylindole) in PBS and incubated for 10 min at RT in the dark. After washing and air-drying, one drop of mounting medium (ProLong Diamond Antifade Mountant, Thermo Scientific) was added, covered with a coverslip, and incubated overnight in the dark. Slides were stored at 4 °C and microscoped within 2 days.

Fluorescence microscopy was performed using a Zeiss Axio Imager Z2 upright microscope with an EC Plan-Neofluar 40x/1.3 DIC M27 objective, and the Zeiss ZEN blue 2 software at MFPL BioOptics (Vienna BioCenter, Austria). The fluorophores were excited with a VIS-LED lamp with filters for DAPI, Alexa 488 and Alexa 594. Four to six regions were imaged per sample and Z-stacks were taken (30-45 slices, 0.250 µm step size).

Data analysis was performed using Fiji^31^. Z-stacks were flattened with the maximum intensity Z-projection and background subtraction was performed using the rolling ball algorithm (10 pixels radius). Automated threshold was applied, using the same for each sample set. The surface coverage (% area) of the *Gardnerella*-probe signal was determined.

### Viability qPCR

At baseline or after treatment, 45–185 µL of samples were transferred into Nonstick, RNase-free microtubes, and centrifuged for 8 min at 7200 × *g*. Cell pellets were treated with PMAxx Dye (Biotium) in the dark following Latka et al.^32^. Briefly, 45 μL of 50 μM PMAxx was added to the cell pellets, incubated 15 min on ice, and exposed to light in a PMA-Lite LED Photolysis Device (Biotium) for 15 min. PMAxx treatment was repeated twice with 2.25 µL of 1 mM PMAxx. Cell pellets were washed with saline, centrifuged for 10 min at 8000 rpm.

gDNA was extracted using the High Pure PCR Template Preparation Kit (Roche) as described above (half the volumes), or the DNeasy 96 Blood & Tissue Kit (Qiagen) following the manufacturer’s instructions with a few changes. For the latter, the protocol for Gram-positive bacteria was followed. Lysis was performed for 90 min, lysates were transferred to DNeasy 96 plate and sealed with AirPore Tape Sheet and centrifuged for 10 min at 3700 rpm. The washing and elution steps were performed on a plate centrifuge. A total of 2 × 40 μL nuclease-free water (Thermo Scientific) were used for elution (at 2560 x *g*) into Elution Microtubes RS. Eluted DNA was aliquoted and stored at −20 °C.

Viability qPCR was performed in a LightCycler 480 (Roche) using EvaGreen qPCR Supermix (Solis Biodyne) with 2 µL extracted DNA as template in a total reaction volume of 10 µL in technical triplicates. qPCR with *Gardnerella* species-specific primer pairs was performed at Microsynth AG (Switzerland) using a LightCycler 480 and SYBR Green qPCR Master Mix (Roche). Primer pair sequences and PCR conditions are shown in Tables S1 and S2, respectively.

### Statistical analyses

Statistical significance was evaluated using ordinary one-way ANOVA followed by Dunnett’s multiple comparison test, using GraphPad Prism 9.5.1, unless stated otherwise.

## Results

### Participant enrollment and characteristics

Between May 2021 and October 2022, a total of 56 women were screened for BV at the Department of Obstetrics and Gynaecology, Medical University of Vienna (Vienna General Hospital), of whom 49 were enrolled in the ex vivo study (the remainder did not meet inclusion/exclusion criteria). Study participant characteristics are provided in Table S3. All enrolled participants had confirmed BV, as diagnosed by a Nugent score ≥7, and 3/4 Amsel criteria with mandatory presence of clue cells. Participants reported to suffer from uncomplicated BV (first-time BV or sporadic episodes, 38–42/49, 78–86%), recurrent BV (≥3 episodes within last 12 months, 9/49, 18%) or refractory BV (chronic, no short-term relief with SOC treatment, 2/49, 4%). A range is given for participants with uncomplicated BV, due to self-reporting and overlap of 6- and 12-months time frames (Table S3).

**Fig. 1:**
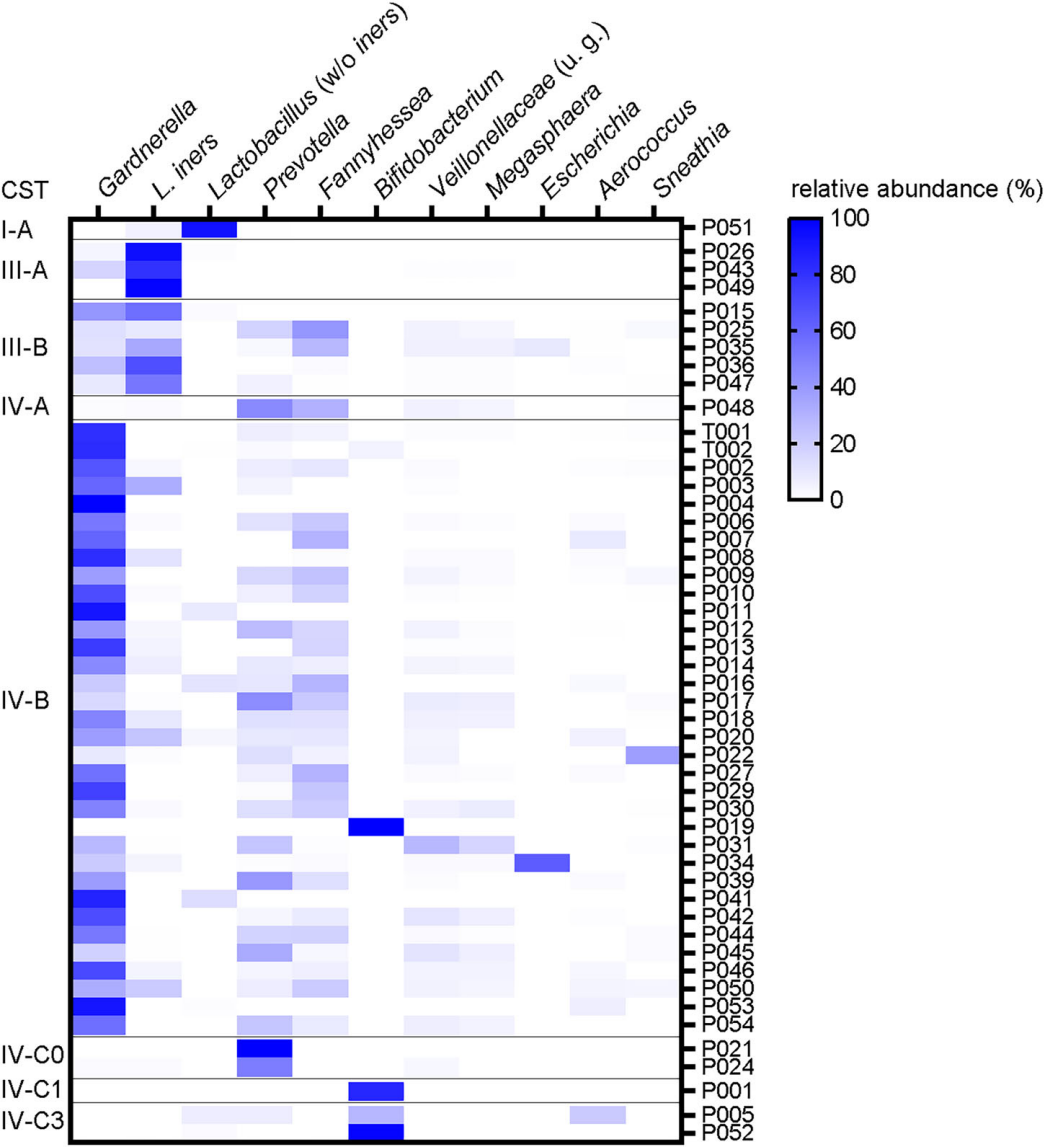
WGS of vaginal samples from study participants. Heatmap illustrating the vaginal microbiomes of 49 vaginal samples of women with BV as determined by WGS. Relative abundance (%) of top ten genera (indicated on top) across samples is shown for individual participants (right). Participant samples are sorted based on their CST classification (left). Bacterial genera are sorted from left to right from most to less overall abundance. *Lactobacillus* genus is split in *L. iners* and other lactobacilli excluding *L. iners*. Family *Veillonellaceae* contains reads for undefined genera.

As assessed by WGS, the prevalent CSTs were IV-B (34/49, 69%), followed by III-B (5/49, 10%) and III-A (3/49, 6%), which is typical for women with BV (Fig. 1, Supplementary Data Set S1). *Gardnerella* was present in 86% (42/49) of participant samples and it was the most abundant genus in 53% (26/49) of samples. *Prevotella*, *Fannyhessea* and *L. iners* were detected in 7% (38/49), 67% (33/49), and 67% (33/49) of participant samples, respectively. Notably, four samples showed very high abundance of *Bifidobacterium* (P005, P001, P052 and P019 with 29%, 85%, 98% and 99.9% relative abundance, respectively) and absence of *Gardnerella*. *Bifidobacterium* is described as producer of lactic acid and was found in some vaginal microbiomes of healthy women^33^. Furthermore, in sample P051 93% abundance of non-*iners* lactobacilli was detected, although all Amsel criteria were met and a Nugent Score of 7 was determined.

### BNT331-EL specifically removes *Gardnerella* in biofilms in vaginal samples from BV-positive women

Vaginal fluid and swab samples were treated with different concentrations of BNT331-EL and a buffer control for 3 h and 19 h and then analyzed by FISH. Samples were hybridized with a probe specific for *Gardnerella* and with a non-specific probe recognizing Eubacteria. Epithelial cell nuclei were stained with DAPI.

**Fig. 2:**
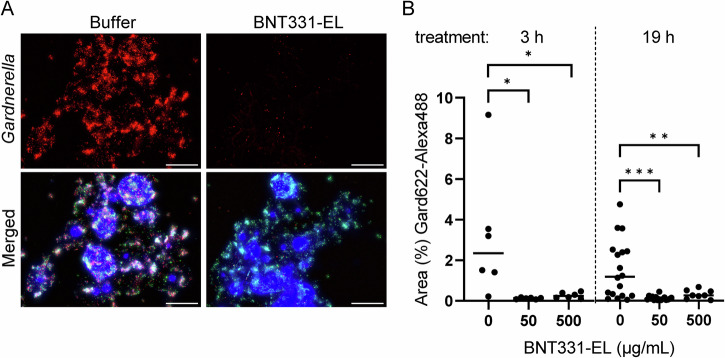
BNT331-EL specifically removes *Gardnerella* biofilm in vaginal fluid assessed by FISH. **A** Representative microscopy images of a vaginal fluid sample (P010) treated for 3 h with 50 µg/mL BNT331-EL or buffer. Human cells are visualized with DAPI (blue), Eubacteria are pseudo-colored in green, *Gardnerella* in red. Background was subtracted using rolling ball subtraction (10 pixels radius) in ImageJ. Scale, 40 µm. Single channel images are provided in Fig. S1A. Images of further participant samples are provided in Fig. S1B. **B** Relative abundance of *Gardnerella* prior to and after treatment with indicated concentrations of BNT331-EL for 3 h or 19 h was determined via the surface coverage of the Gard662-Alexa488 probe signal. Each dot represents the median of 4–6 images per participant sample. Six samples were treated for 3 h, 18/16/8 samples were treated for 19 h with 0/50/500 µg/mL BNT331-EL, respectively. Ordinary one-way ANOVA (p3h = 0.0186; p19h = 0.0002) followed by Dunnett´s multiple comparisons test was performed using GraphPad. *p ≤ 0.05; **p ≤ 0.01; ***p ≤ 0.001. Similar analysis with swab samples is provided in Fig. S2.

As typical for BV, exfoliated human epithelial cells covered with an adherent bacterial biofilm, so called clue cells, were observed in the buffer-treated samples (Fig. 2A, left panel). A large portion of observed bacteria were labeled with the *Gardnerella*-specific probe (pseudo-colored red). After treatment with BNT331-EL, the fluorescence signal for *Gardnerella* was drastically reduced, indicating its killing and removal of the bacterial biofilm. Single fluorescence channels of Fig. 2A are provided in Fig. S1A, representative images from four additional participants are shown in Fig. S1B. To minimize the bias of the readout, we developed a semi-quantitative automated approach that compares the fluorescent areas in the *Gardnerella*-specific channel in the endolysin-treated sample to the buffer-treated aliquot. A statistically significant average reduction of *Gardnerella* in the biofilms was observed after endolysin treatment compared to buffer-treated samples (Fig. 2B). Comparable observations were made in vaginal swab samples (Fig. S2).

### BNT331-EL reduces bacterial growth of *Gardnerella* ex vivo

To better quantify the effects of BNT331-EL on the bacterial load in participant samples, a viability quantitative PCR (qPCR) method was applied, which had previously been optimized for this purpose^32^. Specific primer pairs for species and the genus *Gardnerella*^32,34^, *L. iners*^35^ and *L. crispatus*^36^ were used to quantify the load of these bacteria prior to and after endolysin treatment (Tables S1 and S2 and Fig. S3). Importantly, all samples were treated with the DNA dye PMAxx prior to gDNA isolation to minimize DNA-amplification from dead cells^32,34^.

**Fig. 3:**
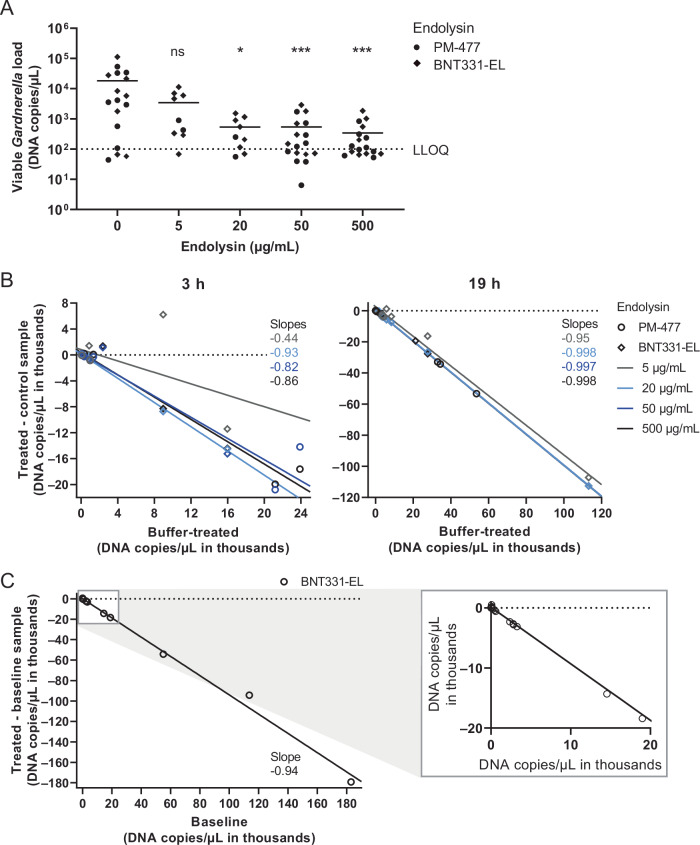
Endolysins reduce *Gardnerella* in participant swab samples in a time- and concentration-dependent manner. **A** Viable *Gardnerella* load (DNA copies/µL) in swab samples after overnight treatment with buffer or endolysin at the stated concentrations determined via viability qPCR. PM-477 and BNT331-EL represent two EL candidates differing by one amino acid with substantially equivalent efficacy (Fig. S4). Individual participant samples were treated with either of the endolysins and are shown as symbols with mean as bar. Each symbol represents the average of qPCR triplicates. *Gardnerella* species-specific (*vaginalis*, *swidsinskii*, *piotii*, *leopoldii*) primer pairs were used, from which the *Gardnerella* load was calculated as sum. Mixed-effects analysis (due to lower number of treated samples at 5 and 20 µg/mL) was performed on log-transformed values followed by Dunnett´s multiple comparison test relative to the buffer-treated aliquots. ns, not significant; *p ≤ 0.05; ***p ≤ 0.001. **B** *Gardnerella* DNA copies/µL in the endolysin-treated samples (3 h or 19 h) subtracted by the load in the respective buffer-treated aliquots are plotted over the *Gardnerella* load in the buffer-treated aliquots. Linear regression across samples treated with the same concentration of either of the endolysins was performed. Coefficient of determination for linear regression after treatment for 3 h: *R*^2^ = 0.29, *R*^2^ = 0.998, *R*^2^ = 0.92, and *R*^2^ = 0.969 for 5, 20, 50 and 500 µg/mL, respectively. Coefficient of determination for linear regression after treatment for 19 h: *R*^2^ = 0.989, *R*^2^ = 0.9998, *R*^2^ = 0.9992 and *R*^2^ = 0.9997 for 5, 20, 50 and 500 µg/mL, respectively. Slopes are stated in the graph and represent relative reduction of the *Gardnerella* load. **C** Thirty vaginal swab samples were treated with 50 µg/mL BNT331-EL for 19 h. The viable *Gardnerella* load was determined via viability qPCR using a *Gardnerella* genus-specific primer pair. DNA copies/µL in treated samples subtracted by the load in respective untreated baseline aliquots are plotted over the load in baseline aliquots. Linear regression across samples was performed, the slope is stated in the graph (*R*^2^ = 0.995). Data points within the gray square are shown as a zoom-in on the right.

First, the optimal endolysin concentration for the treatment was established in a concentration-response study (Fig. 3A, B). Vaginal swabs from 17 participants were treated for 3 h or 19 h with four different endolysin concentrations, followed by viability qPCR using *Gardnerella*-specific primers. Comparison of the viable *Gardnerella* load after endolysin treatment with the viable bacterial load after a mock-treatment (buffer control) showed significant reduction with 20-500 µg/mL, but not with 5 µg/mL endolysin (Fig. 3A). Note that data was pooled from experiments using the endolysins BNT331-EL and PM-477, which only differ in one amino acid. The two endolysins have substantially equivalent lytic activity against *Gardnerella*, and differ only in expression levels and stability (Fig. S4). A high variety of initial *Gardnerella* load (range 10^2^-10^6^ DNA copies/µL) was observed across participant samples, in line with the varying relative *Gardnerella* abundance detected by WGS (see Fig. 1). To evaluate differences in endolysin effectiveness across samples with low or high initial *Gardnerella* load, we plotted the reduction of bacterial load (DNA copies/µL in the buffer-treated controls subtracted from the *Gardnerella* load in the endolysin-treated aliquot) as a function of bacterial load after buffer-treatment (Fig. 3B). Numerically, the treatment efficacy in this analysis is represented by the slope of the linear regression applied for different endolysin concentrations. For example, a slope of –0.998 (99.8% efficacy) for treatment with 20 µg/mL endolysin for 19 h indicated that the growth of *Gardnerella* was ~0.2% of the growth seen in the buffer-treated control. Figure 3B indicates that indeed, both low and high initial loads were effectively inhibited in growth. The overnight treatment resulted in 99.7–99.8% inhibition with 20 µg/mL, 50 µg/mL and 500 µg/mL of endolysin. Five µg/mL was less effective (95%), and this difference was more pronounced when analyzing a shorter treatment period (3 h, 44% efficacy).

The highest endolysin concentration tested (500 µg/mL) did not increase efficacy compared to 50 µg/mL, therefore a concentration of 50 µg/mL was chosen for further experiments.

### BNT331-EL quantitatively reduces *Gardnerella* load in vaginal samples ex vivo

As *Gardnerella* grew in the overnight buffer control in most samples (data not shown), we also evaluated whether treatment with BNT331-EL could reduce the load of viable *Gardnerella* below the baseline value (i.e., before treatment). To this end, a similar experiment as described above was performed with 30 vaginal swab samples, which were all treated with 50 µg/mL BNT331-EL overnight. A baseline sample was processed with viability qPCR to measure the bacterial load before treatment. Figure 3C shows the initial *Gardnerella* load on the horizontal axis and the reduction of the initial load (calculated by subtracting the initial DNA copies/µL from the load after treatment) on the vertical axis. Since the slope of the linear regression on all samples was −0.94, the initial viable *Gardnerella* load was reduced by 94%. A concentration of 50 µg/mL BNT331-EL is therefore sufficient to quantitatively reduce *Gardnerella* independently of the initial load in an ex vivo setting.

### BNT331-EL allows for growth of *L. crispatus* and partially kills *L. iners*

**Fig. 4:**
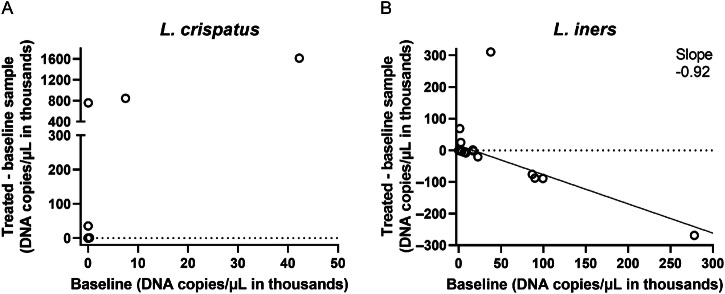
BNT331-EL reduces bacterial load of *L. iners*, but not *L. crispatus.* DNA copies/µL of **A** *L. crispatus* and **B** *L. iners* were determined in vaginal swabs of 30 study participants before (baseline) and after overnight treatment with 50 µg/mL BNT331-EL via viability qPCR. DNA copies/µL in treated samples subtracted by the load in respective untreated baseline samples are shown as a function of the DNA copies/µL at the baseline. Each circle represents one participant sample. **A** Linear regression line is not shown, *R*^2^ = 0.77. **B** Coefficient of determination for the linear regression line is *R*^2^ = 0.39.

To investigate the specificity of BNT331-EL, the same set of 30 samples as shown in Fig. 3C was analyzed by viability qPCR with primers specific for *L. crispatus* and *L. iners*, both at baseline and after treatment (Fig. 4). In contrast to the effect on *Gardnerella*, BNT331-EL did not kill *L. crispatus* when it was present at baseline. In fact, *L. crispatus* was able to proliferate in the presence of BNT331-EL in four samples. Linear regression indicated a low degree of correlation between *L. crispatus* growth during treatment and its presence at baseline (*R*^2^ = 0.7655, regression line not shown), which just indicates that *L. crispatus* grows well in the medium and under the conditions the samples were incubated. Interestingly, *L. iners*, which is a bacterial species associated with an imbalanced vaginome, was substantially reduced by >20,000 DNA copies/µL after treatment compared to baseline in five samples and was increased by >20,000 DNA copies/µLin three samples (Fig. 4B). Averaged across samples, the reduction was 92% based on the slope of the linear regression, however, given the diverging outcome across samples, with a very low *R*^2^ of 0.39.

## Discussion

It is widely accepted that BV is characterized by an imbalance in the vaginal microbiome, which includes a low abundance of beneficial lactobacilli. The etiology of BV is still under investigation, but *Gardnerella* is discussed as one of the key pathogens^6^. In prior publications, *G. vaginalis* was detected in >90% of symptomatic BV patients^3,37,38^, which is in line with the findings presented herein (*Gardnerella* was present in 86% of samples). In a BV study population of South African women, *Gardnerella* was the most abundant genus in 19 out of 56 (34%) patients^3^. This is somewhat lower than in our study population, in which *Gardnerella* dominated in 53% of samples. In another study conducted by Ravel et al. on asymptomatic women, *Gardnerella*-dominant microbiomes were much less prevalent, but *Gardnerella* was still present in 84% of samples taken from participants with NS ≥ 7^2^. These studies indicate that the composition of the vaginal microbiome is complex and varies between study populations. Here, we show that above a threshold concentration of 50 µg/ml, BNT331-EL removes *Gardnerella* irrespective of its abundance at baseline. However, the influence of the microbiome composition at baseline on the potential treatment effect of BNT331 remains to be investigated in clinical trials.

*L. iners* is discussed as a transitional species that may facilitate a shift into the disease state^4,39^. After MTZ treatment, patients often transition into a *L. iners*-dominated microbiome^3^, followed by recurrence. Interestingly, a recent study by Colbert et al. associated some strains of *L. iners* to a failure to respond to cervical cancer treatment, with some genomic distinctions in *L. iners* isolated from cervical cancer patients compared to non-cancer-associated isolates^40^. Differences between *L. iners* strains may also be associated with the distinction between some *L. iners* dominated microbiomes that are stable versus others that transition to the BV state^4,41^. It is therefore noteworthy that BNT331-EL is also active against some *L. iners* strains. We observed that *L. iners* grew in three samples but was substantially reduced in five other samples during endolysin treatment. We did not isolate or further investigate the differences between the responding and not responding *L. iners* strains. Reduction of *L. iners* by the endolysin might ease the regrowth and re-establishment of a stable microbiome by commensal lactobacilli. Consistently, in this study, we observed growth of *L. crispatus* in some of the samples during endolysin treatment, which is in line with the inactivity of the endolysins against commensal lactobacilli reported earlier in an in vitro setting^23^.

Endolysins have been proposed as treatment modalities in bacterial infections since the early 2000s, due in part to their favorable safety profile compared to other new treatment options (reviewed by Murray et al.^42^). Only recently have endolysins been proposed for the treatment of BV^23,43^. This study further substantiates our earlier findings with PM-477 treatment of *Gardnerella* strains and of single or dual species biofilms in vitro^23–25^, but now using clinical samples from BV-positive women to investigate the pharmacodynamics of BNT331-EL ex vivo. In our earlier studies^25^, the minimum biofilm eradication concentration on *Gardnerella* biofilms was <2–32 µg/mL, which implies a minimum effective area under the curve of 768 µg∗h/mL (32 µg/mL ∗ 24 h). This area under the curve is in the same range as the effective area under the curve we determined for *Gardnerella* in this study (20–50 µg/mL ∗ 19 h = 380–950 µg∗h/mL), where a multi-bacterial biofilm in a complex environment was treated.

Strengths of this study include the methods, such as viability-qPCR, which is based on rigorous optimization for this purpose prior to the study^32^. The use of viability qPCR allowed for the assessment of only the viable bacterial load after ex vivo treatment. The use of vaginal samples from women clinically diagnosed with BV to test *Gardnerella*-targeted endolysins added to our understanding of the efficacy of these endolysins in a complex, physiological environment containing multi-species biofilms. Whether the reduction of *Gardnerella* would translate into clinical benefits when BNT331 is applied as a treatment for BV in patients, needs to be tested in interventional clinical trials.

A limitation of our study is that the number of study participants included was rather small. Also, the assessment of the endolysin treatment effect by qPCR limited the quantitation to individual species/genera of bacteria. Therefore, the impact of the removal of *Gardnerella* and *L. iners* on the rest of the bacterial community remains elusive.

In conclusion, we determined the minimum effective concentration and treatment duration of BNT331-EL to quantitatively reduce *Gardnerella* in vaginal samples of women with BV ex vivo. We were able to demonstrate the potential of BNT331-EL as an investigational drug candidate for treatment of BV, supporting further evaluation in a clinical intervention study. A first-in-human trial with BNT331-EL as a vaginal insert was initiated in July 2024 (NCT06469164).

## Supplementary information

Supplementary Figures And Tables

Supplementary Dataset

## Data Availability

All data provided are anonymized to respect the privacy of patients who have participated in the trial, in line with applicable laws and regulations. The raw microbiome sequence data generated in this study have been deposited in the NCBI Sequence Read Archive (SRA) under BioProject accession number PRJNA1274191.

## Abbreviations

BNT331-EL: BNT331-endolysin
BV: bacterial vaginosis
CST: community state type
DAPI: 4’,6-diamidino-2-phenylindole
FISH: Fluorescence in situ hybridization
gDNA: genomic DNA
MTZ: metronidazole
qPCR: quantitative polymerase chain reaction
SOC: standard of care
WGS: whole genome sequencing

## References

[1] Muzny, C. A. & Sobel, J. D. Understanding and preventing recurring bacterial vaginosis: important considerations for clinicians. *Int. J. Womens Health* **15**, 1317–1325 (2023). 37581202 10.2147/IJWH.S383333 PMC10423565

[2] Ravel, J. et al. Vaginal microbiome of reproductive-age women. *Proc. Natl. Acad. Sci. USA* **108**, 4680–4687 (2011). 20534435 10.1073/pnas.1002611107 PMC3063603

[3] Mtshali, A. et al. Temporal changes in vaginal microbiota and genital tract cytokines among South African women treated for bacterial vaginosis. *Front. Immunol.* **12**, 730986 (2021). 34594336 10.3389/fimmu.2021.730986 PMC8477043

[4] Zheng, N., Guo, R., Wang, J., Zhou, W. & Ling, Z. Contribution of Lactobacillus iners to vaginal health and diseases: a systematic review. *Front. Cell Infect. Microbiol.* **11**, 792787 (2021). 34881196 10.3389/fcimb.2021.792787 PMC8645935

[5] Machado, A. & Cerca, N. Influence of biofilm formation by Gardnerella vaginalis and other anaerobes on bacterial vaginosis. *J. Infect. Dis.* **212**, 1856–1861 (2015). 26080369 10.1093/infdis/jiv338

[6] Muzny, C. A. et al. An updated conceptual model on the pathogenesis of bacterial vaginosis. *J. Infect. Dis.* **220**, 1399–1405 (2019). 31369673 10.1093/infdis/jiz342 PMC6761952

[7] Schwebke, J. R., Muzny, C. A. & Josey, W. E. Role of Gardnerella vaginalis in the pathogenesis of bacterial vaginosis: a conceptual model. *J. Infect. Dis.* **210**, 338–343 (2014). 24511102 10.1093/infdis/jiu089

[8] Swidsinski, S., Moll, W. M. & Swidsinski, A. Bacterial vaginosis-vaginal polymicrobial biofilms and dysbiosis. *Dtsch. Arztebl. Int.* **120**, 347–354 (2023). 37097068 10.3238/arztebl.m2023.0090 PMC10412922

[9] Swidsinski, A. et al. An adherent Gardnerella vaginalis biofilm persists on the vaginal epithelium after standard therapy with oral metronidazole. *Am. J. Obstet. Gynecol.* **198**, 97.e91–96 (2008). 10.1016/j.ajog.2007.06.039 18005928

[10] Bradshaw, C. S. & Sobel, J. D. Current treatment of bacterial vaginosis-limitations and need for innovation. *J. Infect. Dis.* **214**, S14–S20 (2016). 27449869 10.1093/infdis/jiw159 PMC4957510

[11] Machado, A., Jefferson, K. K. & Cerca, N. Interactions between Lactobacillus crispatus and bacterial vaginosis (BV)-associated bacterial species in initial attachment and biofilm formation. *Int. J. Mol. Sci.* **14**, 12004–12012 (2013). 23739678 10.3390/ijms140612004 PMC3709769

[12] Kenyon, C., Colebunders, R. & Crucitti, T. The global epidemiology of bacterial vaginosis: a systematic review. *Am. J. Obstet. Gynecol.* **209**, 505–523 (2013). 23659989 10.1016/j.ajog.2013.05.006

[13] Peebles, K., Velloza, J., Balkus, J. E., McClelland, R. S. & Barnabas, R. V. High global burden and costs of bacterial vaginosis: a systematic review and meta-analysis. *Sex. Transm. Dis.* **46**, 304–311 (2019). 30624309 10.1097/OLQ.0000000000000972

[14] Chow, K. et al. Impact of (recurrent) bacterial vaginosis on quality of life and the need for accessible alternative treatments. *BMC Womens Health* **23**, 112 (2023). 36934289 10.1186/s12905-023-02236-z PMC10024842

[15] Kenfack-Zanguim, J. et al. Systematic review and meta-analysis of maternal and fetal outcomes among pregnant women with bacterial vaginosis. *Eur. J. Obstet. Gynecol. Reprod. Biol.* **289**, 9–18 (2023). 37611538 10.1016/j.ejogrb.2023.08.013

[16] Farr, A. et al. Bacterial vaginosis: guideline of the DGGG, OEGGG and SGGG (S2k-Level, AWMF Registry No. 015/028, June 2023). *Geburtshilfe Frauenheilkd.* **83**, 1331–1349 (2023). 37928409 10.1055/a-2169-8539 PMC10624544

[17] Abbe, C. & Mitchell, C. M. Bacterial vaginosis: a review of approaches to treatment and prevention. *Front. Reprod. Health* **5**, 1100029 (2023). 37325243 10.3389/frph.2023.1100029 PMC10264601

[18] Muzny, C. A. & Sobel, J. D. The role of antimicrobial resistance in refractory and recurrent bacterial vaginosis and current recommendations for treatment. *Antibiotics* **11**, 10.3390/antibiotics11040500 (2022). 10.3390/antibiotics11040500 PMC9024683 35453251

[19] Sobel, J. D. et al. Suppressive antibacterial therapy with 0.75% metronidazole vaginal gel to prevent recurrent bacterial vaginosis. *Am. J. Obstet. Gynecol.* **194**, 1283–1289 (2006). 16647911 10.1016/j.ajog.2005.11.041

[20] Johnston, W. et al. In vitro bacterial vaginosis biofilm community manipulation using endolysin therapy. *Biofilm* **5**, 100101 (2023). 36655001 10.1016/j.bioflm.2022.100101 PMC9841237

[21] Muzny, C. A. & Schwebke, J. R. Pathogenesis of bacterial vaginosis: discussion of current hypotheses. *J. Infect. Dis.* **214**, S1–S5 (2016). 27449868 10.1093/infdis/jiw121 PMC4957507

[22] Fischetti, V. A. Bacteriophage endolysins: a novel anti-infective to control Gram-positive pathogens. *Int. J. Med. Microbiol.* **300**, 357–362 (2010). 20452280 10.1016/j.ijmm.2010.04.002 PMC3666336

[23] Landlinger, C. et al. Engineered phage endolysin eliminates Gardnerella biofilm without damaging beneficial bacteria in bacterial vaginosis ex vivo. *Pathogens* **10**, 10.3390/pathogens10010054 (2021). 10.3390/pathogens10010054 PMC7830407 33435575

[24] Castro, J. et al. Exploiting the anti-biofilm effect of the engineered phage endolysin PM-477 to disrupt in vitro single- and dual-species biofilms of vaginal pathogens associated with bacterial vaginosis. *Antibiotics* **11**, 10.3390/antibiotics11050558 (2022). 10.3390/antibiotics11050558 PMC9137943 35625202

[25] Landlinger, C. et al. Preclinical data on the Gardnerella-specific endolysin PM-477 indicate its potential to improve the treatment of bacterial vaginosis through enhanced biofilm removal and avoidance of resistance. *Antimicrob. Agents Chemother.* **66**, e0231921 (2022). 35416708 10.1128/aac.02319-21 PMC9112913

[26] McCracken, J. M. et al. Animal models and alternatives in vaginal research: a comparative review. *Reprod. Sci.* **28**, 1759–1773 (2021). 33825165 10.1007/s43032-021-00529-y PMC8204935

[27] Miller, E. A., Beasley, D. E., Dunn, R. R. & Archie, E. A. Lactobacilli dominance and vaginal pH: why is the human vaginal microbiome unique?. *Front. Microbiol.* **7**, 1936 (2016). 28008325 10.3389/fmicb.2016.01936 PMC5143676

[28] Morrill, S., Gilbert, N. M. & Lewis, A. L. Gardnerella vaginalis as a cause of bacterial vaginosis: appraisal of the evidence from in vivo models. *Front. Cell Infect. Microbiol.* **10**, 168 (2020). 32391287 10.3389/fcimb.2020.00168 PMC7193744

[29] France, M. T. et al. VALENCIA: a nearest centroid classification method for vaginal microbial communities based on composition. *Microbiome* **8**, 166 (2020). 33228810 10.1186/s40168-020-00934-6 PMC7684964

[30] Swidsinski, A. & Loening-Baucke, V. In *Fluorescence In Situ Hybridization (FISH): Application Guide* (ed. Liehr, T.) 531–543 (Springer, 2017).

[31] Schindelin, J. et al. Fiji: an open-source platform for biological-image analysis. *Nat. Methods* **9**, 676–682 (2012). 22743772 10.1038/nmeth.2019 PMC3855844

[32] Latka, A. et al. Optimization of propidium monoazide qPCR (Viability-qPCR) to quantify the killing by the Gardnerella-specific endolysin PM-477, directly in vaginal samples from women with bacterial vaginosis. *Antibiotics (Basel)* **11**, 10.3390/antibiotics11010111 (2022). 10.3390/antibiotics11010111 PMC8773202 35052988

[33] Freitas, A. C. & Hill, J. E. Quantification, isolation and characterization of Bifidobacterium from the vaginal microbiomes of reproductive aged women. *Anaerobe* **47**, 145–156 (2017). 28552417 10.1016/j.anaerobe.2017.05.012

[34] Balashov, S. V., Mordechai, E., Adelson, M. E. & Gygax, S. E. Identification, quantification and subtyping of Gardnerella vaginalis in noncultured clinical vaginal samples by quantitative PCR. *J. Med. Microbiol.* **63**, 162–175 (2014). 24200640 10.1099/jmm.0.066407-0

[35] De Backer, E. et al. Quantitative determination by real-time PCR of four vaginal Lactobacillus species, Gardnerella vaginalis and Atopobium vaginae indicates an inverse relationship between L. gasseri and L. iners. *BMC Microbiol.* **7**, 115 (2007). 18093311 10.1186/1471-2180-7-115 PMC2233628

[36] Byun, R. et al. Quantitative analysis of diverse Lactobacillus species present in advanced dental caries. *J. Clin. Microbiol.* **42**, 3128–3136 (2004). 15243071 10.1128/JCM.42.7.3128-3136.2004 PMC446321

[37] Munch, M. M. et al. Gardnerella species and their association with bacterial vaginosis. *J. Infect. Dis.*10.1093/infdis/jiae026 (2024). 10.1093/infdis/jiae026 PMC11272073 39052736

[38] Srinivasan, S. et al. Bacterial communities in women with bacterial vaginosis: high resolution phylogenetic analyses reveal relationships of microbiota to clinical criteria. *PLoS ONE* **7**, e37818 (2012). 22719852 10.1371/journal.pone.0037818 PMC3377712

[39] Petrova, M. I., Reid, G., Vaneechoutte, M. & Lebeer, S. Lactobacillus iners: friend or foe?. *Trends Microbiol.* **25**, 182–191 (2017). 27914761 10.1016/j.tim.2016.11.007

[40] Colbert, L. E. et al. Tumor-resident Lactobacillus iners confer chemoradiation resistance through lactate-induced metabolic rewiring. *Cancer Cell* **41**, 1945–1962.e1911 (2023). 37863066 10.1016/j.ccell.2023.09.012 PMC10841640

[41] France, M., Alizadeh, M., Brown, S., Ma, B. & Ravel, J. Towards a deeper understanding of the vaginal microbiota. *Nat. Microbiol.* **7**, 367–378 (2022). 35246662 10.1038/s41564-022-01083-2 PMC8910585

[42] Murray, E., Draper, L. A., Ross, R. P. & Hill, C. The Advantages and challenges of using endolysins in a clinical setting. *Viruses* **13**, 10.3390/v13040680 (2021). 10.3390/v13040680 PMC8071259 33920965

[43] Arroyo-Moreno, S., Cummings, M., Corcoran, D. B., Coffey, A. & McCarthy, R. R. Identification and characterization of novel endolysins targeting Gardnerella vaginalis biofilms to treat bacterial vaginosis. *NPJ Biofilms Microbiomes* **8**, 29 (2022). 35440653 10.1038/s41522-022-00285-0 PMC9018826

