# Supplementary material for: Endolysin selectively kills Gardnerella ex vivo in vaginal samples from women with bacterial vaginosis
Source: NPJ Biofilms Microbiomes. 2025 Aug 12;11:161. doi: 10.1038/s41522-025-00764-0 (PMC12344050; doi:10.1038/s41522-025-00764-0)
Supplement: Supplementary file 1 — Supplementary Figures And Tables [file 41522_2025_764_MOESM1_ESM.pdf]

SUPPLEMENTARY FIGURES AND TABLES

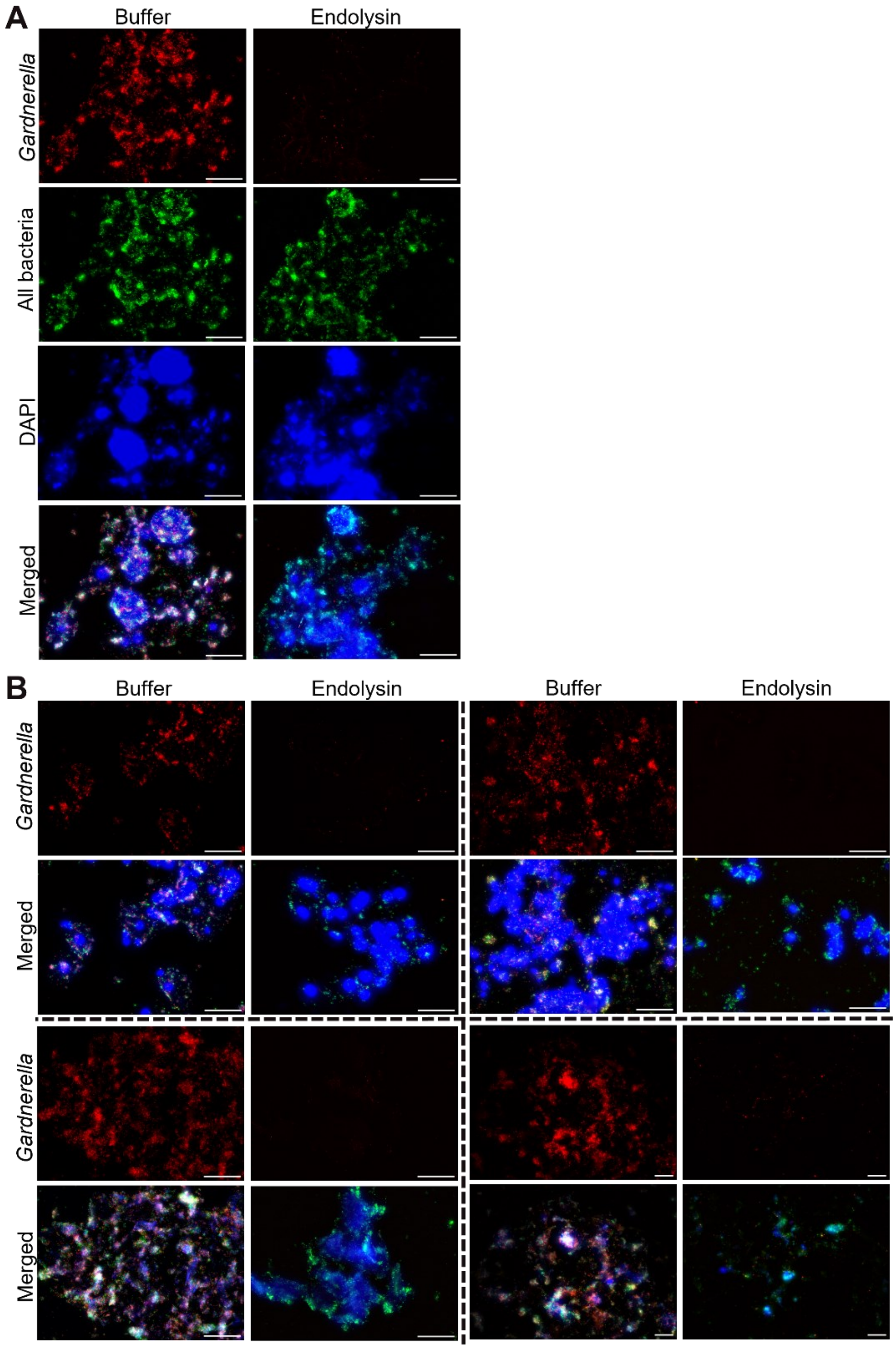

Supplementary Figure S1. FISH analysis of BNT331-EL-treated samples

Vaginal fluid samples from BV-positive women were treated with BNT331-EL or incubated with buffer. Samples were stained with fluorescent probes specific for *Gardnerella* (red) or bacteria in general (green). Nuclei were stained with DAPI. A) Split channels of the image provided in Figure 2A, P010. B) Four additional analysed samples (left to right: upper panel: P013, P015; lower panel: P014, T001) are shown. Samples were treated for 3 h with 50 µg/mL BNT331 EL, except for T001, which was treated with 500 µg/mL BNT331.

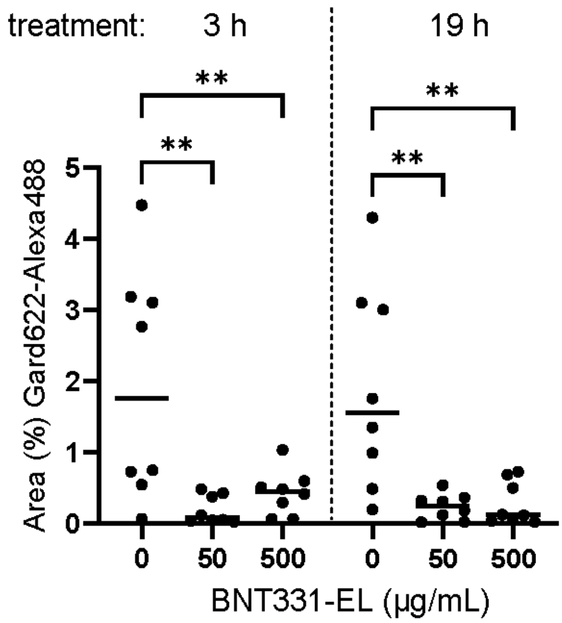

Supplementary Figure S2. BNT331-EL specifically kills *Gardnerella* biofilm in vaginal swabs

Abundance of *Gardnerella* prior to and after treatment with indicated concentrations of BNT331-EL for 3 h or 19 h was determined via the surface coverage of the Gard662-Alexa488 probe signal. Ordinary one-way ANOVA ($p_{3\ h}$=0.0028; $p_{19\ h}$=0.0010) followed by Dunnett´s multiple comparisons test was performed using GraphPad. *, p≤0.05; **, p≤0.01.

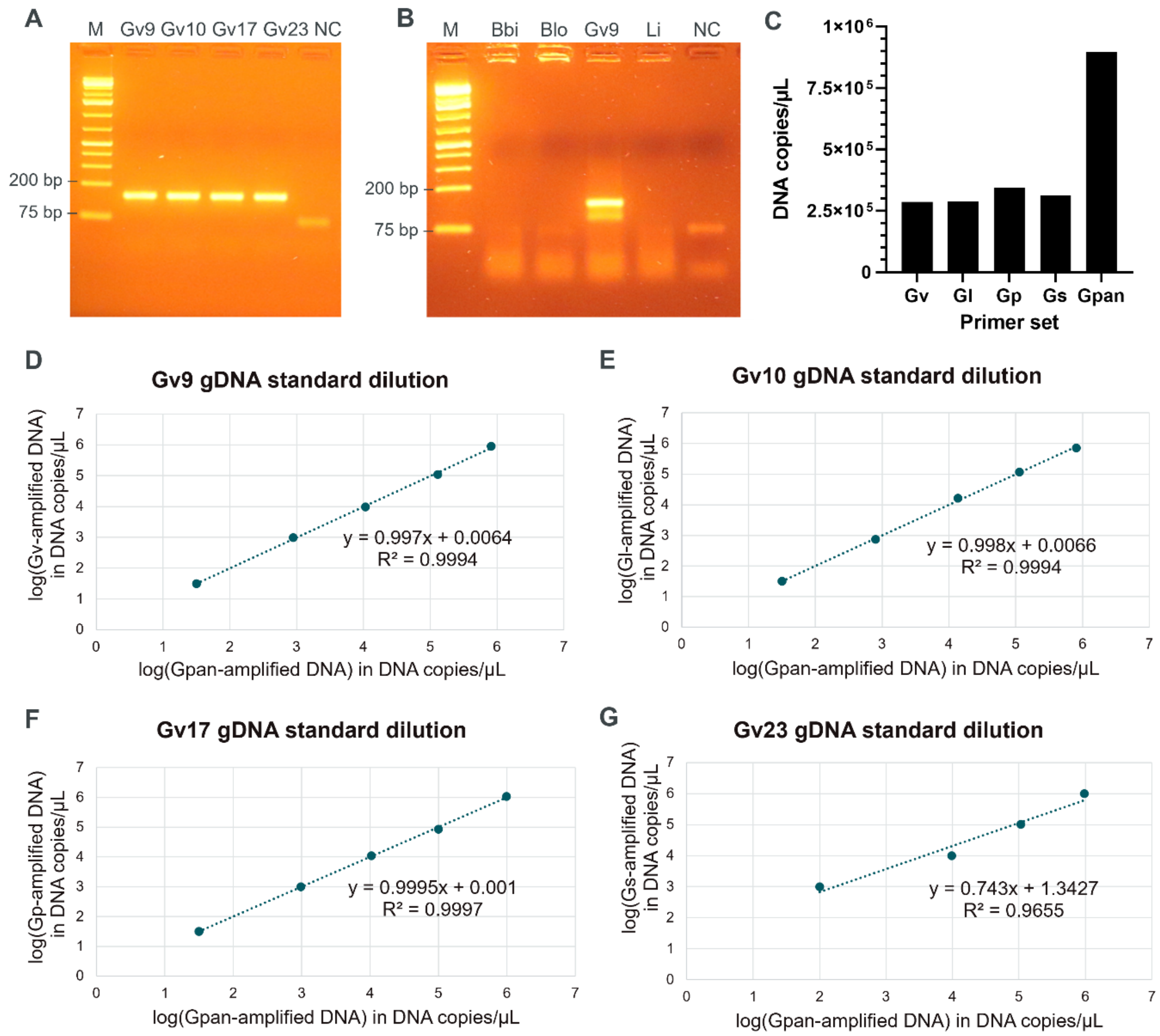

Supplementary Figure S3. Specificity testing of *Gardnerella* genus-specific primer pair for qPCR.

A-B) Specificity testing of the *Gardnerella* genus-specific primer pair (Gpan) against **A)** gDNA from *G. vaginalis* (Gv9*), G. leopoldii* (Gv10), *G. piotii* (Gv17) and *G. swidsinskii* (Gv23) and **B)** gDNA of *Bifidobacterium bifidum* (Bbi), *B. longum* (Blo), Gv9 *and L. iners* (Li) showed amplification of DNA from *Gardnerella* species, but not of other tested strains. Gpan-amplified amplicon size corresponds to 149 bp. NC, negative control. **C)** *Gardnerella* DNA copies/µL detected by qPCR using species-specific primer pairs[1] for *G. vaginalis* (Gv), *G. leopoldii* (Gl), *G. piotii* (Gp), and *G. swidsinskii* (Gs), or the Gpan primer set. Specificity of species-specific primer pairs and efficiency of the genus-specific primer pair was confirmed. The sample was composed of equal concentrations (2.5×10^5 DNA copies/µL) of gDNA of the four *Gardnerella* species Gv9, Gv10, Gv17, and Gv23. **D-G)** Correlation analysis of Gpan vs. *Gardnerella* species-specific (Gv, Gl, Gp and Gs) primer sets when amplifying Gv9, Gv10, Gv17, and Gv23 gDNA, respectively, standard dilutions. Equation of the linear regression and R^2 values are stated.

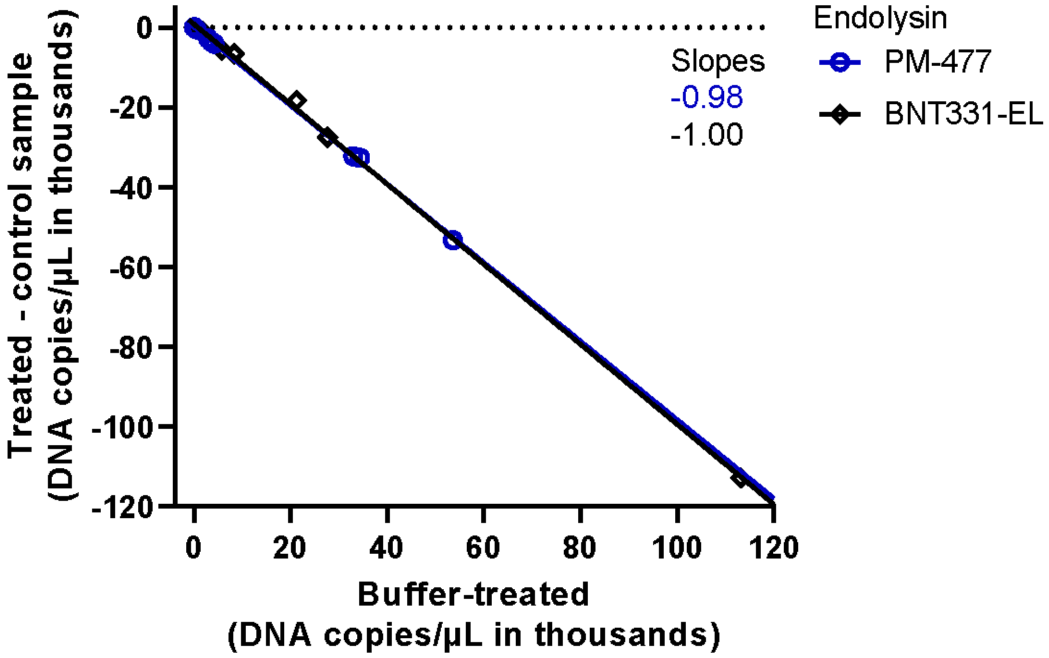

Supplementary Figure S4. Functional equivalence of BNT331-EL and PM-477

Gardnerella DNA copies/µL in the endolysin-treated (50 µg/mL) samples (overnight) subtracted by the respective buffer-treated samples are plotted over the *Gardnerella* load in the buffer-treated samples. Linear regression across participants samples treated with endolysins PM-477 (n=9, R^2=0.9995) or BNT331-EL (n=8, R^2=0.9993) was performed. Slopes are stated in the graph.

Supplementary Table S1

Species/genus	Primer sequence	Reference
Gardnerella	TCCCAACCCCAACTCACGATCTT	Balashov et al., 2014[2]
	RCGCAAACCAACRATCTCAACTGG	Balashov et al., 2014[2]
G. vaginalis	TATTATAACTAAAGCTGCTG	Latka et al., 2022[1]
	TCGCCACTATAGTCG	Latka et al., 2022[1]
G. swidsinskii	ATTTAGTTAGATATTTGGCAA	Latka et al., 2022[1]
	ATAGTCATATATTCCGCGC	Latka et al., 2022[1]
G. piotii	AGCTGCTTACGATTATAGT	Latka et al., 2022[1]
	TTACTCATTCTAAGCTTAATAG	Latka et al., 2022[1]
G. leopoldii	GATACTGCACTGTATCGA	Latka et al., 2022[1]
	CAGTATCAATACCAGCC	Latka et al., 2022[1]
Lactobacillus crispatus	AGCGAGCGGAACTAACAGATTTAC	De Backer et al., 2007[3]
	AGCTGATCATGCGATCTGCTT	De Backer et al., 2007[3]
Lactobacillus iners	GTCTGCCTTGAAGATCGG	De Backer et al., 2007[3]
	ACAGTTGATAGGCATCATC	De Backer et al., 2007[3]

Supplementary Table S1. Primer sequences used for viability qPCR

Supplementary Table S2

**Primer pair**	**Condition**
Gardnerella	Initial denaturation: 95 °C, 12 min<br>40 cycles: 15 s at 95 °C, 20 s at 60 °C, 25 s at 72 °C<br>Melting analysis: 55-95 °C with 2.5 °C/s ramp rate
L. iners & *L. crispatus*	Initial denaturation: 95 °C, 12 min<br>40 cycles: 15 s at 95 °C, 40 s at 57 °C, 30 s at 72 °C<br>Melting analysis: 55-95 °C with 2.5 °C/s ramp rate
Gardnerella species-specific	Initial denaturation: 95 °C, 5 min<br>40 cycles: 15 s at 95 °C, 30 s at 56 °C, 30 s at 72 °C<br>Melting analysis: 55-95 °C with 2.5 °C/s ramp rate

Supplementary Table S2. Conditions for qPCR.

Supplementary Table S3

	Value ± SD	Range
**Patients involved (n)**	49	
**Ethnicity (n)**		
African	2	
Asian	2	
Caucasian	42	
Middle East	3	
**Age (years)**	32.2±6.8	20 to 49
**Nugent Score**	7.6±0.6	7 to 10
**Vaginal pH**	5.1±0.2	4.7 to 5.5
**Pregnant**	26	
**Previous episodes of BV in last 6 months (n)**		
None	19	
1-2	23	
>2	3	
Permanent	2	
Unknown	2	
**in last 12 months (n)**		
None	19	
1-2	16	
3-5	8	
>5	1	
Permanent	2	
Unknown	3	
**in last 3 years (n)**		
None	10	
1-2	17	
3-5	15	
>5	3	
**Permanent**	2	
**Unknown**	2	

Supplementary Table S3. Study population characteristics (previous episodes of BV were self-reported by the study subjects).

Supplementary References

1 Latka, A. *et al.* Optimization of Propidium Monoazide qPCR (Viability-qPCR) to Quantify the Killing by the Gardnerella-Specific Endolysin PM-477, Directly in Vaginal Samples from Women with Bacterial Vaginosis. *Antibiotics (Basel)* **11** (2022). https://doi.org/10.3390/antibiotics11010111

2 Balashov, S. V., Mordechai, E., Adelson, M. E. & Gygax, S. E. Identification, quantification and subtyping of Gardnerella vaginalis in noncultured clinical vaginal samples by quantitative PCR. *J Med Microbiol* **63**, 162-175 (2014). https://doi.org/10.1099/jmm.0.066407-0

3 De Backer, E. *et al.* Quantitative determination by real-time PCR of four vaginal Lactobacillus species, Gardnerella vaginalis and Atopobium vaginae indicates an inverse relationship between L. gasseri and L. iners. *BMC Microbiol* **7**, 115 (2007). https://doi.org/10.1186/1471-2180-7-115
